# Supplementary material for: Factors for the Early Revision of Misdiagnosed Tuberculosis to Lung Cancer: A Multicenter Study in A Tuberculosis-Prevalent Area
Source: J Clin Med. 2019 May 17;8(5):700. doi: 10.3390/jcm8050700 (PMC6571679; doi:10.3390/jcm8050700)
Supplement: Supplementary file 1 [file jcm-08-00700-s001.pdf]

Table S1. The demographics of patients according to the timing to obtain pathology proof.

	**Early Obtain Pathology (*n* = 26)**	**Late Obtain Pathology (*n* = 19)**	***p* Value**
Age, year	65.6±13.6	66.7±13.2	0.748
Male sex	19 (73%)	16 (68%)	0.734
Microbiology			
AFS *			0.686
positive	5 (19%)	4 (21%)	
negative	20 (77%)	15 (79%)	
Culture *			0.114
NTM	2 (8%)	6 (32%)	
negative	10 (39%)	6 (32%)	
Radiographic pattern			0.959
nodular	16 (62%)	12 (63%)	
consolidation	9 (34%)	6 (32%)	
pleural effusion	1 (4%)	1 (5%)	
Lesion size, cm	5.8±2.8	2.7±1.1	<0.001
Bilateral	5 (19%)	1 (5%)	0.081
Cavitation	5 (19%)	5 (26%)	0.572
Miliary pattern	5 (19%)	0	0.043
Pleural effusion amount			0.303
<1/3 hemithorax	3 (12%)	0	
1/3-2/3 hemithorax	3 (12%)	2 (11%)	
>2/3 hemithorax	0	1 (5%)	
The cause for TB suspicion			0.626
AFS (+)	5 (19%)	7 (37%)	
Pathology suspicion	1 (4%)	1 (5%)	
Radiological suspicion	15 (58%)	8 (42%)	
Clinical suspicion	5 (19%)	3 (16%)	
Anti-TB treatment	18 (69%)	19 (100%)	0.008
Days to diagnosis revision	42.2±37.3	78.6±46.4	0.006
Days to pathology obtained	9.8±10.7	111.9±83.7	<0.001

Abbreviation: AFS, acid-fast smear; TB, tuberculosis; NTM, nontuberculous mycobacteria; * One case an 21 cases did not have examinations for AFS and mycobacteria culture, respectively.

Study Period:

The cases screening period:

National Taiwan University Hospital, Taipei: Jan 2009 – June 2018

National Taiwan University Hospital, Yun-Lin Branch: Jan 2014 – Dec 2018

National Yang-Ming University Hospital: Jan 2007 – Dec 2018

Far Eastern Memorial Hospital: Jan 2007 – Dec 2018

E-Da Hospital: Jan 2014 – Dec 2018
